# Enhanced cryopreservation of MSCs in microfluidic bioreactor by regulated shear flow

**DOI:** 10.1038/srep35416

**Published:** 2016-10-17

**Authors:** Akalabya Bissoyi, Arindam Bit, Bikesh Kumar Singh, Abhishek Kumar Singh, Pradeep Kumar Patra

**Affiliations:** 1 Department of Biomedical Engineering, National Institute of Technology, Raipur, India; 2 Department of Biochemistry, University of Allahabad, Allahabad, India; 3 Department of Biochemistry, Pt. JNM Medical College, Raipur, India

## Abstract

Cell-matrix systems can be stored for longer period of time by means of cryopreservation. Cell-matrix and cell-cell interaction has been found to be critical in a number of basic biological processes. Tissue structure maintenance, cell secretary activity, cellular migration, and cell-cell communication all exist because of the presence of cell interactions. This complex and co-ordinated interaction between cellular constituents, extracellular matrix and adjacent cells has been identified as a significant contributor in the overall co-ordination of tissue. The prime objective of this investigation is to evaluate the effects of shear-stress and cell-substrate interaction in successful recovery of adherent human mesenchymal-stem-cells (hMSCs). A customized microfluidic bioreactor has been used for the purpose. We have measured the changes in focal-point-adhesion (FPAs) by changing induced shear stress inside the bioreactor. The findings indicate that with increase in shear stress, FPAs increases between substrate and MSCs. Further, experimental results show that increased FPAs (4e-3 μbar) enhances the cellular survivability of adherent MSCs. Probably, for the first time involvement of focal point interaction in the outcome of cryopreservation of MSCs has been clarified, and it proved a potentially new approach for modification of cryopreservation protocol by up-regulating focal point of cells to improve its clinical application.

Mesenchymal stem cells (MSCs) play a vital role in clinical science, differentiation of MSCs lead to formation of multiple mesenchymal lineages including muscle, cartilage, fat, and bone^1^. Moreover, MSCs are useful for epithelial differentiation/re-epithelization and wound healing. MSCs seeded scaffolds serve as a balanced platform for cellular and tissue growth^2^. In spite of several advantages in the field of tissue engineering, one of the major limitations is the long-term storage and transportation of engineered sample during transplantation procedure. The cryopreservation of these cells and scaffold based constructs could be a possible approach to address the issue. In near future, patient specific tissue engineered products will be an integral approach for clinical applications that involve evaluation of cryopreserved process with practical terms of synthesis such as manufacturing process must accommodate successful viability of large number of cells in the matrix or in the scaffold^3^.

In cryopreservation method, parity is given to maintain the integrity of membranous structure of the cell sheets, tissue and laboratory produced organ in bioethical manner. Several strategies for cryopreservation include ultra rapid freezing and thawing, controlled-rate freezer, freezing with non-penetrating polymers, vitrification and equilibrium freezing^4^. Moreover, there are several other important factors to be considered for the successful cryopreservation viz., composition of cryopreservation medium, nature of cryoprotective agents (CPAs), screening procedure, thawing procedure and intrinsic susceptibility of the cells to the freezing damage^5^. The frequently applied protocol for cryopreservation of cell suspension comprises of slow cooling of cells and rapid thawing in presence of high concentration of dimethyl sulphoxide (DMSO) and animal derived product^6^.

Responses of complex cell system and simple cell suspension to cooling, warming and dehydration have not yet been taken into account, which often results in poor quality of post-preservation^7^. Although, many cells can be successfully cryopreserved in suspension, but the cells in monolayer are subjected to cryoinjury during cryopreservation. The cell-matrix and cell-cell junction and associated organization of the cellular cytoskeleton in monolayered cells are more prone towards cryoinjury as compared to the isolated suspended cells^8^. The cryopreservation associated phase changes often results into osmotic stress that lead to damage to top sites of cellular interaction. In addition, the cells adhered to substrate are more susceptible to intracellular ice formation (ICIF) as compared to suspended cells in absence of intracellular interaction^9^.

Thus, the engineered cells and tissues can be prepared efficiently for clinical purposes only after successful cryopreservation of monolayer cells. It also provides benefit to cell-based assay as it eliminates batch-to batch variation, inoculation process, and cellular expansion from frozen vial of cells, and thus shortened the pathways between storage and implication procedures^10^.

Macromolecular assembly associated with surrounding extracellular matrix (ECM) through integrin receptor is known as focal adhesions (FAs). FAs serve as the linkage between actin and ECM that integrate environmental signals into adhesion mediated signaling networks, which further regulate reorganization of actin cytoskeleton and thereby changes morphology, behavior, and the cellular fate.

This phenomenon is usually associated with every cell types ranging from prokaryotes to multi-cellar organisms. The force-sensitive FAs proteins may undergo structural re-arrangement upon exposure to mechanical forces and enzymatic modifications. These factors and attributed phenomena alter the binding preferences, in comparison to other FA associated proteins, leading to re-modulation of protein associated with FAs. There are growing body of evidences showing the link between the mechanical properties of the extracellular environment and cellular decision-making mechano-transduction processes^11^,^12^. Our current understanding of adhesion-mediated environmental sensing is still fragmentary and several design principles have emerged from experiments. The biological responses elicit due to either force transmitted via adhesion proteins (Integrin, Cadherin) of cell membrane or in presence of fluid shear stress being transmitted directly to the cell membrane and the receptors present on surface^13^,^14^.

Material strength of the cells, sensing mechanism of external forces, and transmitting those forces to nucleus are provided by the cytoskeleton structure. When endothelial cells are exposed to shear stress less than one Pascal, a chain of morphological and transcriptional alteration triggers^15^, and nutrophiles may occur in response to even smallest shear stress^16^. Externally applied stresses or traction forces are transmitted through focal adhesion (FA) receptors and are distributed throughout the cell, causing conformational changes, phosphorylation events, and enzymatic activities. In addition, individual mechno-sensing proteins may change their binding affinity. FAs being larger multi protein complexes provide a mechanical link between ECM and cytoskeleton contraction machinery. When FAs subjected to pulling forces, they self-assembled to elongate, and dissociated on release of forces^17^. A thermodynamic model can be used to understand, mechano-sensitivity of FAs. A molecular aggregate, when exposed to cooling forces tends to align and grow in the orientation of applied force by additional molecules incorporation. This behavior of FAs helps spreading and movement of cells along substrate. Mechano-sensitivity of FAs can be assessed by observing adhesion sites when exposed to mechanical force either by means of contractile machinery of the cell or due to external perturbation. The effect of external forces on cell-matrix contacts is believed that FAs act as mechano-sensors, converting force into biochemical reaction^18^.

Herein, we believe that shear stress induced focal adhesion point (FPA) plays an important role in cryopreservation. In order to validate the hypothesis, we investigated ability of MSCs following cryopreservation in DMSO and trehalose, and also evaluated *in vivo* efficiency of trehalose-cryopreserved MSCs seeded on microchannel. In particular, our results indicate that low shear stress induced focal point enhances cell viability. Furthermore, the assessment of cryopreserved MSCs seeded on microchannel was performed with both quantitative and qualitative methods.

## Results

In the present study, MSCs derived from umbilical cord blood (UCB) of human were cryopreserved by slow freezing method using DMSO, trehalose, ectoine and catalase as cryoprotective agents. The effect of shear stress on outcome of cryopreservation was determined by seeding MSCs within the microfluidic based cell observation chamber.

MSCs were cultured within the device in MSCs-specific growth medium throughout the channel until the considerable confluency was reached. The effect of shear stress was evaluated on proliferation of cells. The shear stress particularly within the range from 0.1 to 0.4 dyn/cm^2^ was produced by different flow rate and its effect on outcome of cryopreservation was evaluated over time. After 8 days of treatment with shear stress within microchannel, the cultured MSCs were cryopreserved using controlled rate freezer (CRF).

### Numerical analysis

**Figure 1:**
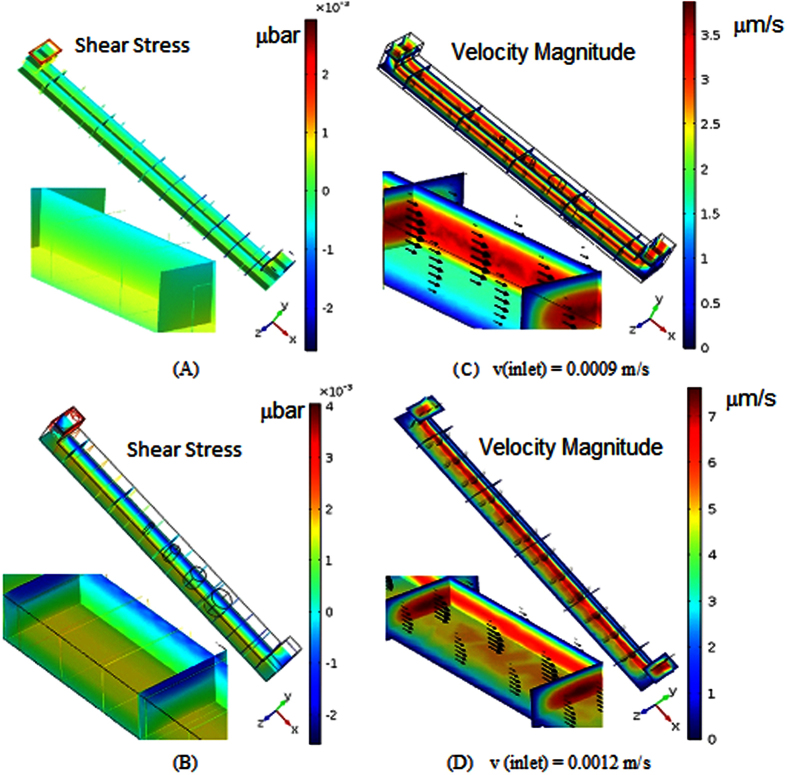
(**A**,**B**) Contour plot of Shear- stress distribution within micro-channel at different flow rates; (**C**,**D**) Contour plot of Velocity magnitude of cell-media composites in micro-channel at three different inlet flow rates.

Transport phenomena of culture-media (containing solute particles) through microchannel results in development of flow-induced shear-stress. Since, the inlet velocity was varied from 0.0009 m/s to 0.0015 m/s, shear stress distribution inside the microchannel was also varying from 0.002 μbar to 0.004 μbar (as shown in Fig. 1). The shear stress on wall near the inlet of the vessel was observed at maximum, whereas within microchannel, its magnitude was found increasing with the depth of channel. At the same time, the gradients in shear stress distribution along the height of the microchannel increases with increased flow-rate of the cellular media (Fig. 1A,B). Sustainability of constant shear stress at different depth of the vessel is found consistent. It can be verified from velocity contour plot (Fig. 1C,D). Consistency of a constant velocity magnitude along the axis of the microchannel is observed at different height of the microchannel. Surprisingly, the gradient in velocity magnitude has been observed while flow-rate at the inlet of the channel is lowest, and reverse phenomena occurred while flow-rate is maximum in microchannel.

### Flowcytometry analysis of MSCs phenotype

**Figure 2:**
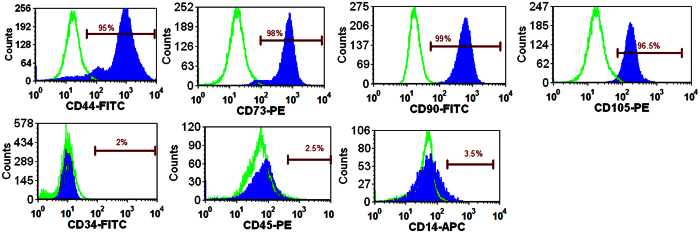
Flow-cytometric analysis showing expression of positive Mesenchymal stem cells (MSC) markers CD44 (95%), CD73 (98%), CD90 (99%) and CD105 (96%) as well as negative MSC markers CD34 (1.2%), CD45 (2%) and CD14 (3.5%).

The effect of induced shear stress on cryopreservation outcome of UCB-derived MSCs phenotype was evaluated using flowcytometry analysis. Flowcytometry analysis revealed that the fresh and cryopreserved MSCs are positive for CD90, CD44, CD105 and CD73 while negative for CD14, CD34 and CD45 (Fig. 2). It indicates that cryopreserved MSCs have retained similar pattern of expression of cell surface markers as of MSCs at 4^th^ passage of growth. Our observations confirm that shear stress and process of cryopreservation (freezing and thawing) did not affect the phenotypes of MSCs.

### Effect of shear stress on MSCs focal point

Focal adhesion points (FPAs) act as locus for adhering cells and mediate the ability of cells to sense and adapt to the mechanical properties of its microenvironment. We studied whether shear stress played distinct role in regeneration of FPAs.

**Figure 3:**
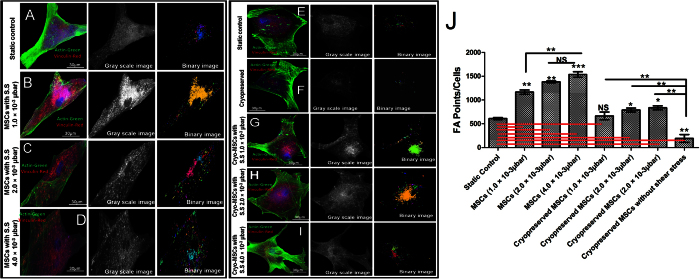
Vinculin associated focal point adhesions: Distribution of actin filaments and vinculin in human mesenchymal stem cells, showing cytoskeletal organization. Actin filament, vinculin and nucleus are pseudocolor in green, red, and blue respectively. (**A**) 8^th^ day, MSCs seeded in microchannel without any shear stress. (**B**) 8^th^ day MSCs seeded in microchannel under 1 × 10^−3^ μbar. (**C**) 8^th^ day MSCs seeded in microchannel under 2 × 10^−3^ μbar. (**D**) 8^th^ day MSCs seeded in microchannel under 4 × 10^−3^ μbar. (**E**) Static Control (**F**) MSCs seeded in microfluidic device with cryopreservation treatment. Cryopreserved MSCs after 8 days of (**G**) 1 × 10^−3^ μbar, (**H**) 2 × 10^−3^ μbar, (**I**) 4 × 10^−3^ μbar shear stress inside microchannel. (**J**) Quantitative difference in focal points in MSCs at various shear stresses compare to static control. (NS: > 0.05, ** < 0.01, *** < 0.005).

After seeding MSCs within the microfluidic cell observation chamber, the MSCs were cultured within the device until the significant confluency was reached by flowing MSC growth medium throughout the chamber. The effect of shear stress was investigated as the cells proliferated. The shear stress was induced by contact flow using syringe pump. Specifically, the effect of different flow-rates, which resulted in shear stresses ranging from 1 to 4.0 × 10^−3^ μbar, on cellular focal point was observed over time. After 8 days in microfluidic culture, the long axis of the MSCs aligned preferentially along the flow direction at this shear stress (Fig. 3).

MSCs were cultured in micro-channel and we found that FPAs (as in form of expression: vinculin) responded more readily as compared to the static control (Fig. 3J). The expression of vinculin was found significantly higher when MSCs were exposed to fluidic plane with shear-stress. Since, MSCs under the influence of shear stress showed significantly higher number of FPAs, we had investigated how shear stress associated FPAs responded in cryopreservation at different parametric settings. The presence of FPAs was investigated not only quantitatively by vinculin-actin staining but also using image segmentation with Matlab. The analysis of fluorescent micrographs (Fig. 3J) shows that the number of FPAs were significantly reduced.

After 1 day time period, cellular focal point of adhesion was quantified by measuring the cells with respect to the 3 different shear stress values (1, 2.0 and 4.0 × 10^−3^ μbar). Under the influence of shear stress, MSCs in regions of the microfluidic device displayed increased expression of focal point with increasing flow-rate, indicated that both shear stresses are necessary for increase expression of the focal point in MSCs. The qualitative measurement of FPA was done at different shear stresses (Fig. 3). In addition, the average FPA was also measured for different shear stresses (Fig. 3I). The results show that the presence of shear is sufficient to induce FPA.

### Effects of shear stress on cell adhesion after cryopreservation

**Figure 4:**
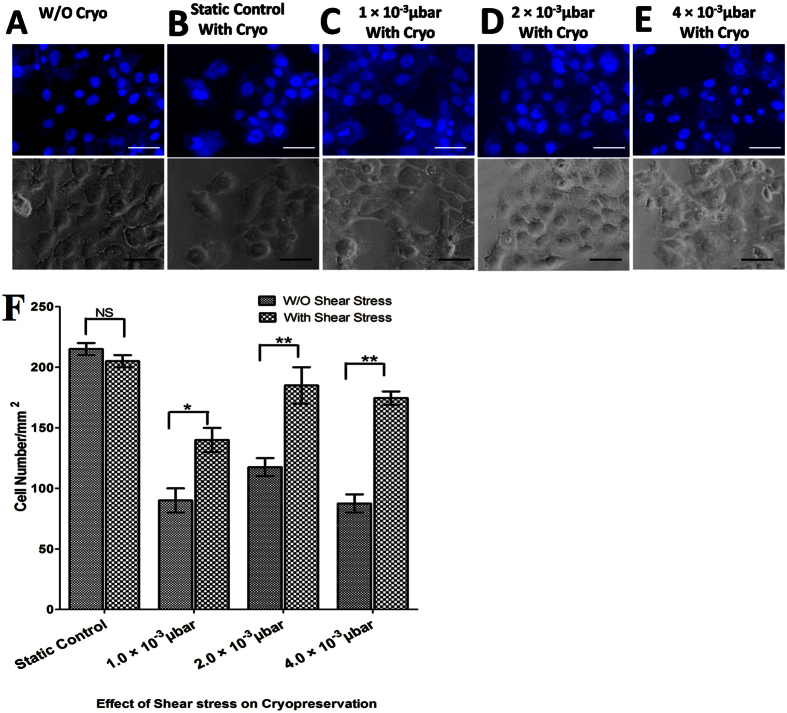
(**A**–**E**) Phase contrast and DAPI staining images of MSCs. MSCs were grown with or without shear stress. Representative images of (**A**) MSCs without cryopreservation, (**B**) MSCs with cryopreservation-static control. Cryopreserved MSCs with shear stress (**C**) 1 × 10^−3^ μbar (**D**) 2 × 10^−3^ μbar (**E**) 4 × 10^−3^ μbar. (**F**) bar plot image of cell number per mm^2^ (NS: > 0.05, ** < 0.01, *** < 0.005).

Apart from maintaining the phenotypes, MSCs should have the ability to sustain the effect of shear stress and freeze thaw cycles. MSCs were seeded in a confluent state and subjected to different shear stresses (1, 2.0 and 4.0 × 10^−3^ μbar) followed by freezing and further thawing after 7 days to evaluate the competency of the protocol. The outcomes of direct thawing and subsequent culture of these cells in microchannels are shown in Fig. 4. The results showed the significant decrease in attachment of MSCs after freeze thaw cycle inside microfluidic culture subjected to zero shear stress (static control). The results also show that the cell attachment did not significantly decrease with increased induced shear stress. In contrast, these exposed regions differed from each other in angle of alignment, resulting in no preferential alignment for the overall sample. The cells were spread and grown following 8 days as shown in Fig. 4. Two days after thawing, cells in microchannels were reached 80% to 90% confluence as shown in Fig. 4.

### Viability of MSCs after cryopreservation

The cell survival, long-term storage and maintenance of functional integrity of MSCs are essential processes, if they are to be banked and used for clinical applications. Reductions in cell viability and functional capacity may have implications for the therapeutic application of tissue engineered constructs^19^. Therefore, in this study, cell viability of MSCs was evaluated in presences of various shears stress and at zero without any induced shear stress (static control). Interestingly, MSCs with induced shear stress showed better cellular viability as compared to static control.

**Figure 5:**
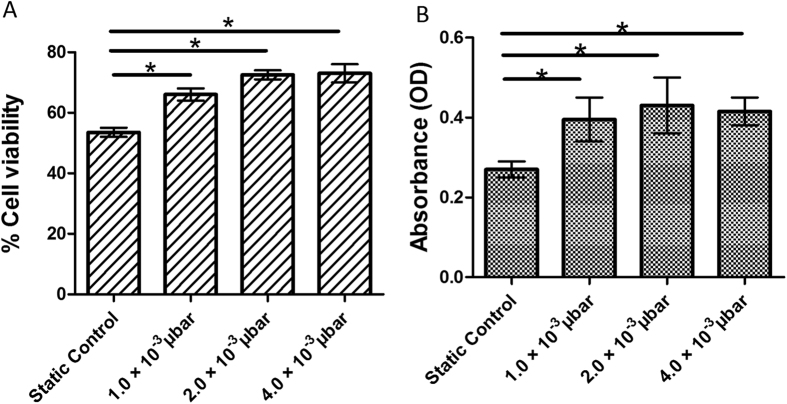
(**A**) Bar plot image of immediate post-thaw viability of MSCs inside microchannel measured by trypan blue. (**B**) Bar plat image of viable MSCs 24 h after post thaw, as assessed by the MTT assay. (NS: > 0.05, ** < 0.01, ***).

Furthermore, the result of cellular viability was further confirmed by MTT assay after 24 hr of cell culture. The MTT data also showed the similar pattern of cell viability tests as shown in trypan blue dye exclusion assay (Fig. 5A) immediately after cryopreservation.

### Effect of shear stress on MSC differentiation

**Figure 6:**
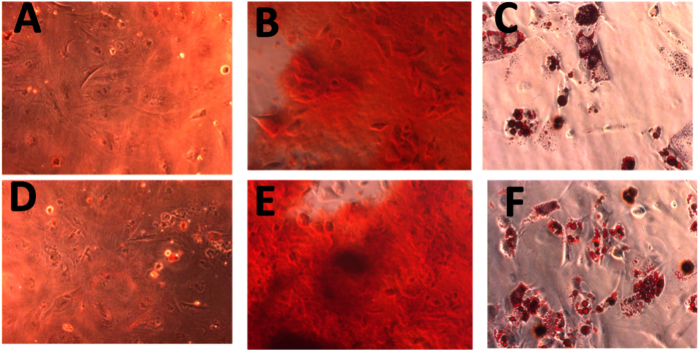
Representative images for differentiation potential of MSCs seeded inside microchannel. Multilineage differentiation of shear induced cryopreserved MSCs showing differentiation potential towards both osteogenic and adipogenic lineages. Adipogenic oil droplet formation and osteogenic calcium nodule formation was determined by Oil Red O and alizarin red assays respectively.

It has been reported that cryopreservation and induced shear stress may influences osteogenic and adipogenic potential of MSCs by up-regulating or down regulating the expression of osteogenic and adipogenic marker genes. To provide an insight into molecular changes that may occur following cellular freezing, we investigated the effect of cryopreservation on MSCs-derived differentiating lineage specific marker gene expression profile. To evaluate osteogenic and adipogenic potential of MSCs after cryopreservation, we did Oil-red-O, Alizarin Red staining and gene expression analysis. Upon adipogenesis, fresh and cryopreserved MSCs under the influence of induced shear stress showed the formation of lipid droplets which were positively stained with Oil red O (Fig. 6)^20^.

Similarly, upon osteogenesis, calcium deposits were stained with Alizarin Red. Several dark red regions, after the onset of osteogenic differentiation, were also observed which indicate the deposition of abundant of calcium in fresh MSCs and cryopreserved MSCs. Thus, the data confirmed the maintenance of osteogenic potential of MSCs after the treatment with shear stress and following cryopreservation. These results also confirmed that cryopreservation maintained the capability of MSCs to undergo adipogenesis while under the treatment of shear stress.

### Effects of cryopreservation on MSCs stemness

**Figure 7:**
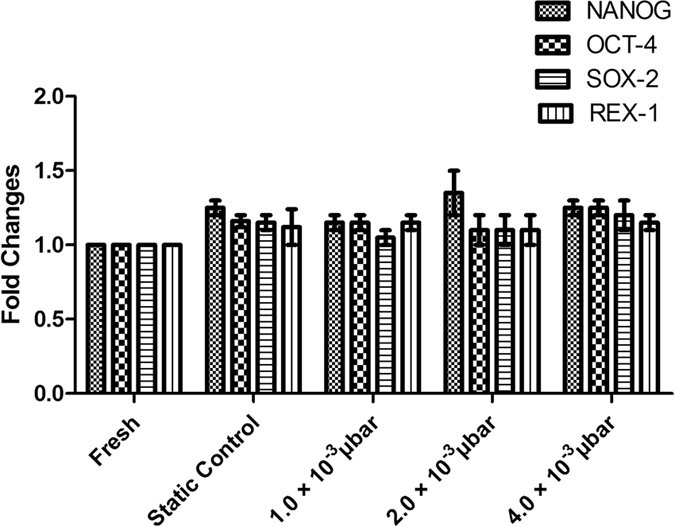
Determination of stemness of MSCs seeded inside microchannel. No significant higher expression level of stemness markers (NANOG, OCT-4, SOX-2 and REX-1) were observed in cryopreserved MSCs in various shear stress compared to fresh and static control.

Stemness is a typical characteristic of MSCs, and it plays an important role in regulating plasticity potential and self-renewal of MSCs. It has been reported that knockdown of REX-1, NANOG, OCT-4, and SOX-2 significantly inhibits proliferation of cell and multipotency of MSCs. To determine the expression of markers of stemness in cryopreserved MSCs under the influence of shear stress, we performed quantitative Real-Time PCR. The cryopreserved MSCs expressed significantly higher (p < 0.05) level of stemness genes such as REX-1, NANOG, OCT-4, and SOX-2 as compared to freshly isolated MSCs (Fig. 7).

However, in this study, both cryopreserved MSCs and fresh MSCs showed similar level of expression of stemness markers, compare to normal proliferation and differentiation potential. Therefore, the cryopreserved MSCs could be able to maintain their stemness. The results thus signified that the induction of controlled shear stress does not influence the differentiation and proliferation capabilities of MSCs.

## Discussion

Cryopreservation of stem cells has been traditionally performed by suspending cells in freezing media. However, the successful therapeutic application of MSCs relies on the development of an appropriate cryopreservation protocol that ensures preservation of tissue engineered construct or in simple form of monolayer culture with desired cell viability, metabolic activity and differentiation ability of MSCs upon storage^21^,^22^. Moreover, it has been well documented that the interaction signal such as cell-cell and cell-ECM greatly influence the behavior of stem cells within the tissue engineered construct^23^. Thus, it is crucial to elucidate the role of the bio-physical elements of the cell-ECM interaction to better preserve stem cells and potentiate their differentiation capacity^24^.

The present study was undertaken to investigate how shear stress induced by fluid movement could influence the cryopreservation outcomes of MSCs. For the purpose, we have used Polydimethylsiloxane (PDMS) based microchannel which were connected with syringe pump, therefore allowing independent control of the fluid flow-rate, permitting to evaluate different shear rate on different properties of MSCs. Particularly in this study, the shear stress rate was equal for every point in microchannel, which was achieved by keeping constant flow-rate. Human derived fibronectin in phosphate buffer saline (PBS) was chosen to facilitate the cellular attachment to the glass bottom of the microchannels^25^. Moreover, in our previous studies, we formulated a freezing medium containing trehalose, ectoine and catalase and very promising results were obtained with MSCs and MSCs-seeded tissue engineered constructs. In this study, the work has been further extended to investigate the effects of these natural osmolytes with low concentration of DMSO as CPA solution for the cryopreservation of UCB-derived MSCs in microchannel.

The formation of intracellular ice between substrate and cell membrane during hypothermia and freezing is another important factor that causes membrane damage leading to loss of viable cell during and after freezing^26^. The focal adhesion points between cell membrane and substrate are significant to overcome this damage by reducing the space between them^27^. The fluidic shear stress acts as a potential inducer of focal point, and thus the shear stress was adopted to evaluate its effectiveness in efficient cryopreservation of MSCs in the present study.

The proliferation capability of MSCs on different substrates was tested prior to evaluation of similar interaction during cryopreservation. The human derived fibronectin was found to retain stemness of the cells in undifferentiated state when it was used as matrix basement membrane. We monitored the stem cell markers up to 4^th^ passage of UCB-derived cultured MSCs and our results showed that all the MSCs were able to express more than 97% of MSCs-specific positive markers and less than 2% hematopoietic stem cell specific positive markers.

The improvement in cell growth after post thaw culture is only achievable by proper cell attachment. During the cryopreservation of cells-seeded on microfluidic device, it is critical for the MSCs to remain attached to the surface of devices as cell viability and growth require the attachment to a solid surface^28^,^29^,^30^ and cryopreservation itself induces the detachment of the adhered cells from the substratum^31^. Figure 4 shows the number and morphology of MSCs cultured on device before and after cryopreservation using natural osmolytes and proportionate amount of DMSO as CPA and frozen at the cooling rate of 1 °C/min. The morphological observations showed that the spindle shape of MSCs is found to be efficiently regulated by induced shear stress in micro channels. Furthermore, the result shows a significant decrease in number of attached cells after cryopreservation which further remained consistent with outcomes of the study conducted by cryopreservation of bovine corneal endothelial monolayer^32^. Moreover, the number of MSCs with or without induced shear stress shows significant difference in their attached cell number. It is expected that induced shear stress may changes the physiological behavior, such as cell-cell and cell-matrix interactions of attached MSCs. The investigation on the effect of induced shear stress on cryopreservation outcome was further extended. Our data also confirmed that the viability and metabolic rate of MSCs significantly varies 24 h after post thaw when treated with induced shear stress.

An improvement in cell viability and metabolic activity of MSCs under induced shear stress might be due to enhanced cell-ECM attachment and shear stress induced local focal point in the cells^33^,^34^. The organization of focal point distribution is a main component in cell-ECM interaction and it plays a vital role in cryopreservation of adherent cells to maintain cell attachment and cell function. An accumulation of focal point complex de-polymerization indicates the role of FPA complex in outcomes of cryopreservation^35^. Hence, we investigated the role of FPAs during cryopreservation of MSCs adhered to microchannel for 8 days. The MSCs were stained with F-actin and vanculin to identify F-actin and FPAs complex organization. As shown in the Fig. 3, shear stress is found to induce more focal points throughout cell boundary compared to MSCs without treatment with shear stress^36^,^37^.

**Figure 8:**
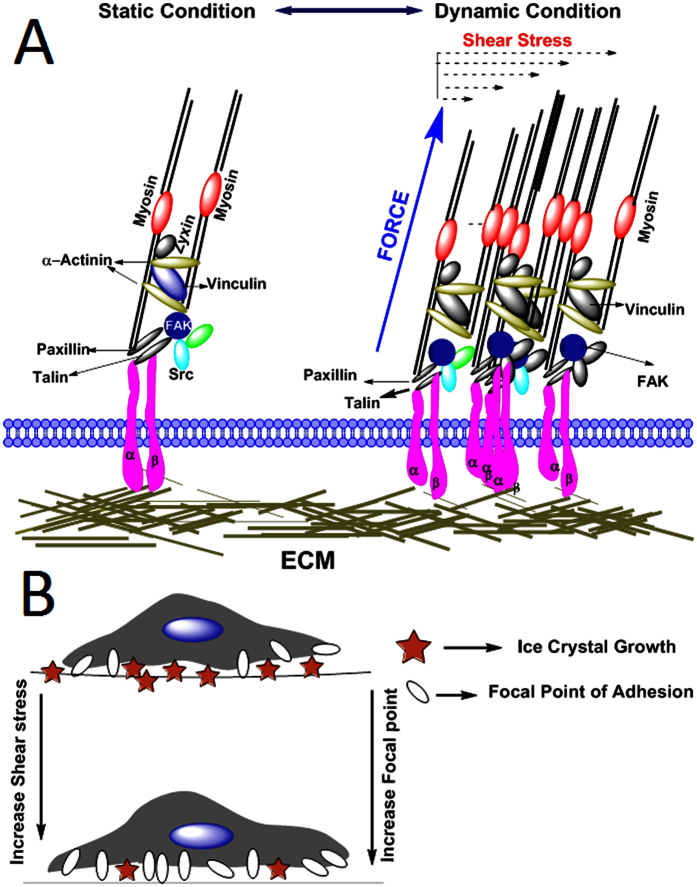
Schematic representation of cell substrate interaction in presence of shear stress. (**A**) With increase in shear stress focal adhesion point increases. (**B**) Schematic representation showing with increase in number of focal point decreases the intracellular space between cell-ECM, results in lesser ice formation in intracellular space.

When stress applied to the cytoskeleton of cells during cryopreservation by virtue of intracellular ice formation beyond the threshold, focal points can withstand and cell eventually dies due to F-actin trans-membrane signaling. In addition, the less number of FPAs were found in MSCs adhered to the substrate of microchannel after cryopreservation. In contrast, adhered MSCs, in presence of shear stress induced FPA, display low density of FPAs before and after cryopreservation. Strikingly, MSCs without shear stress showed less focal point density and altered cell morphology in rounded shape. The result reveals the possible role of FPAs during induced shear stress of MSCs in efficient recovery after cryopreservation. It has been observed that shear stress can induce differentiation of MSCs to particular lineage such as neuron or cartilage^38^,^39^,^40^, hence we monitored the stemness and plasticity potential into different lineages before and after cryopreservation under defined shear stress conditions. Our data showed that the stemness and differentiation potential of the MSCs were not affected with fluid flow induced shear stress. The finding implies that shear stress of low magnitude did not influence the stem cells property^41^. Overall, the present study confirmed the existence of a connection between induced shear stress and outcomes of cryopreserved MSCs through FPAs and shear stress induced increased expression of vinculin. With increase in shear stress, FPAs also increase through specific transcription factors involved in stem cell fate decision^42^. Nevertheless, more detailed studies are still required to determine the underlying molecular and physical mechanism. In addition to the shear stress induced focal point, we have also hypothesized that with increase in number focal point the interstitial space between ECMs and cell membrane tends to decreases which may reduce the chances of intercellular ice formation during the process of cryopreservation (Fig. 8). In summary, it can be concluded that MSCs attached to a particular substrate can effectively cryopreserved, if subjected to lower shear stress for particular duration of time.

## Conclusion

Our findings conclude that the fluid flow can induce shear stress inside microchannel which further induces the expression of focal point in cells. The expression profile of focal point complex can be efficiently altered by changing the shear stress. The altered focal point complex due to shear stress induces changes in interaction pattern at cell-cell and cell-ECM interface that play crucial role in determining the cell survivability. In addition, the enhanced cell viability and cell recovery after cryopreservation are also attributed due to the better attachment of cell to ECM through focal point. As a conclusion, the interaction pattern at the FPAs plays vital role in determining the MSCs viability in cryopreservation.

The outcomes from this investigation could enhance the establishment of standardized cryopreservation protocol of human MSCs attached to the substrate for future clinical applications. Accomplishment of the above benefits can only be fully operational when the structure and function of cell are preserved during the process of cryopreservation. It could be critical in the case of complex and sensitive systems such as embryonic stem cells, engineered cells and tissues, monolayer cell cultures, three-dimensional cell aggregates and biosynthetic constructs.

### Materials and Methodology

All the experimental methods and protocols described in this section were carried out in accordance with the approved guidelines of Institutional ethical committee of National Institute of Technology, Raipur (NITR), and Pt. Jawaharlal Nehru Memorial Medical College, Raipur (Pt. JNMMCR), India. All the experimental protocols were approved by NITR and Pt. JNMMCR. Informed consent was obtained (both in regional and National languages) from all the participating volunteers for the collection of umbilical cord blood.

### Chemicals and antibodies

Dulbecco’s phosphate buffered saline (DPBS), Dulbecco’s modified eagle medium (DMEM), fetal bovine serum (Hi-FBS), antibiotic (Penicillin-Streptomycin) solution, and 0.25% Trypsin/EDTA solution used were procured from Gibco (BRL, USA). Phalloidin-Alexa Fluor 488 conjugate was purchased from Invitrogen, USA. Anti-Vinculin antibody was purchased from Sigma-Aldrich (St Louis, MO, USA). Ficoll hypaque solution (Hisep LSM 1073), adipogenic differentiation media, Triton X-100, formaldehyde, MTT assay, adipogenic and osteogenic differentiation media were purchased from Hi-Media labs, India. Growth factors- EGF and bFGF, BD FACS lysis buffer, CD34-PE, CD14-PE, CD44-FITC, CD45-PE, CD73-PE, CD90-FITC, and CD105-PE were purchased from BD pharminogen (Becton Dickenson, San Jose, CA). Oil red O, Alizarin red S, Safranin O were procured from Sigma-Aldrich (St Louis, MO, USA). All the tissue culture plastic wares were purchased from BD falcon.

### Numerical modeling of problem

Numerical study was conducted for modeling the flow of cell-media in microchannels. Dimensions of the micro-channel were fixed as 2250 × 150 × 150 micron. Convective and diffusive equations of flow were solved using COMSOL Multiphysics (USA).

### Governing equations and boundary conditions

The flow rate at the inlet is simplified to plug-steady flow, and it is varied from 0.5 × 10^−6^ m/s to 8 × 10^−6^ m/s^43^. The Reynolds number corresponding to the flow is given by Equation 1:


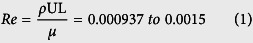


where ρ is the fluid density (1000 kg/m^3^), *U* is a characteristic velocity of the flow (0.5 × 10^−6^ m/s to 8 × 10^−6^ m/s), μ is the fluid viscosity (1.2 mPa⋅s) and *L* is a characteristic dimension of the device (2250 μm). Since the Reynolds number is significantly less than 1, the Creeping-flow interface is used to define the transport phenomena of biological media (cell-culture media) through micro channel. The modified form of Navier-Stokes equations for incompressible flow of media in micro-channel at ceasing Re can be represented by the following Equations (2) and (3):


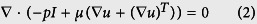



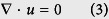


where u is the axial velocity (m/s) of media, and *p* represents the pressure (Pa). Mixing of solute particles in the media while transporting through the symmetric channel-integrated wells involves species (nutrients) at relatively low concentrations compared to the solvent media. Interaction of solute molecules with the solvent media can be defined as diffusive transport, and it obeys Fick’s law. Therefore, the mass-balance equation for the solute is represented by the Equation 4:


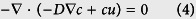


where c is its concentration (mol/m^3^) and diffusion coefficient of the solute (m^2^/s)is represented by D. At wall, no-slip boundary condition is considered, where zero gauge pressure was considered at outlet of microchannel.

### Microfludic Device Fabrication

#### Characterization of pseudo-grayscale with backside diffused light lithography (pGS BDLL)

pGS BDLL can produce multi-level micro-fluidic devices at low-cost set-up. This technique was first established by Lai *et. al*.^44^. Isotropic pattern of binary opaque and transparent 2250 by 150 μm rectangular patterns were created at low-resolution photo-mask on transparent paper to produce long microchannel with SU-8 negative photo-resist.

#### Characterization of pGS BDLL (Methodology)

pGS masks were developed using COMSOL CAD module, and printed by CAD-Art Services (Bandon, OR) at 18,000 DPI. Individual pseudo-gray areas were created using an isotropic pattern of binary opaque and transparent 2250 by 150 μm rectangular patterns. With such patterns, areas of 100% and 80% transparent were printed on the photo-mask for pGS BDLL.

#### Device fabrication and channel characterization

**Figure 9:**
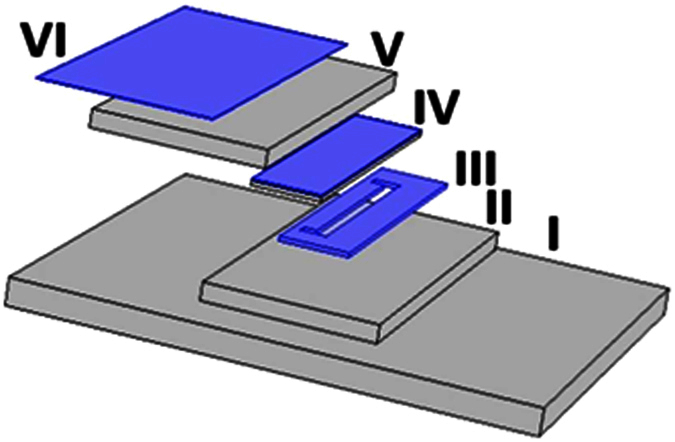
(Schematic of Backside Diffused Light Lithography (BDLL): (**I**) represents the UV trans-illuminator from which UV light passes through (**II**) glass substrate and (**III**) pGS photo-mask (a mask on which the pattern of the micro-channel is drawn) to expose (**IV**) SU-8 substrate to produce micro-channel of exactly same pattern by regulating the amount of energy allowed to pass through the spatial region of pGS photo-mask. (**V**) Quartz Glass is used to cover SU-8 coated glass, whereas, (**VI**) a tinted film is used at the top of the set-up for preventing it from exposed on light.

pGS masks with varying percent of transparent areas were used for BDLL. Negative photo-resist (SU-8 2075 and 2025, MicroChem, Newton, MA) was deposited on cover-glass slides at 100 μm with a spin coater. Thickness and different graded transparent conditions in each case was exposed to 8 mW/cm^2^ UV illuminator (816A Ultraviolet Trans-illuminator, Fisher Scientific, Waltham, MA). The resultant PDMS channel heights were determined using light microscopy as well as fluorescence microscopy of channels filled with 0.01% fluoresce-in as previously described as shown in Fig. 9.

#### Shear stress inside microchannels

Shear stress was determined using fluorescent microscopic examination of the cells near the wall of microchannels. After inducing dynamic stress on cells for 8 days, cells were fixed with 2% paraformaldehyde for 1 hour. The permeabilization of cells was achieved with 0.5% Triton X-100 in PBS for 15 min. Non-specific binding of fixed cells was restricted by blocking the cells with bovine serum albumin (2% w/v) for 20 minutes. For focal point detection, anti-vinculin antibody (1:200), actin filaments (Alexa-Fluor phalloidin 488) and nuclear DNA (DAPI, 300 nM) were used respectively. The fluorescent images of actin filaments, focal adhesions and nuclei were captured using Leica TCS SP5 Super continuum Confocal Microscope (Leica, Germany).

#### Isolation and culture of hUCB derived MSCs

In order to perform the tests, umbilical cord blood (UCB) was collected with prior approval of Institutional Ethical Committee (Pt. Jawaharlal Nehru Memorial Medical College, Raipur, India), and written informed consent of volunteers. The MSCs were isolated following density gradient method^45^. In brief, UCB was diluted in the ratio of 4:1 with RPMI 1640 media and 30 ml of diluted cord blood sample was layered over 20 ml of Ficoll hypaque solution slowly along the sides of the centrifuge tube followed by centrifugation at 430 × g for 30 min at 4 °C. From the distinct three layers, the buffy coat containing MNCs was separated. Then, 1x lysis buffer (1:9 dilution of 10x stock buffer in distilled water) was added to the MNCs sample, and incubated for 10 min at room temperature. The cell suspension was centrifuged at 200 × g for 30 min at 4 °C. The pellet was washed to remove lysis buffer and other impurities with the help of repeated centrifugation (with D-PBS) at 200 × g for 10 min each time. Finally, the pellet obtained was re-suspended in expansion medium consisting of DMEM, 10% FBS, 2 mM glutamine, growth factors such as EGF, bFGF and 1% antibiotic-antimycotic solution, and are plated at density of 1 × 10^6^ cells/ml. The medium was changed twice weekly. Cells were trypsinized when significant confluency was reached and sub-cultured up to fourth passages before further experimentation.

#### Morphological and immuno-phenotypic characterization

The morphological characteristics of cultured UCB-derived MSCs were assessed by microscopic observation. The expression pattern of stem cell specific surface antigen was evaluated using flowcytometer BD FACSJazz™, Becton Dickinson (USA). The trypsinized fourth passaged cells (5 × 10^5^cells per sample) were stained with human monoclonal antibodies against positive (CD44, CD73, CD90, CD105) and negative (CD34, CD45 and CD14) surface markers of MSCs.

#### Cryopreservation experiment

**Figure 10:**
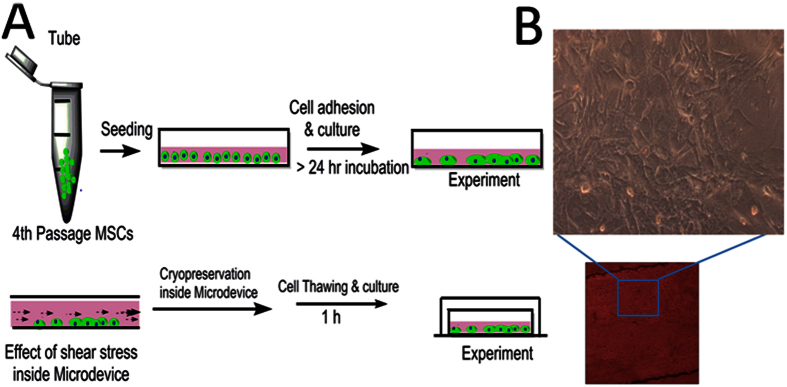
Illustration of cryopreservation strategy used for MSCs inside microfluidic chamber (**A**) Schematic representation of cell seceding and subsequent cryopreservation protocol (**B**) Phase contrast image of MSCs inside microfluidic chamber and it enlarge image with 10X magnification obtained from confocal microscope.

The freezing medium was composed of 2.5% DMSO, trehalose, ectoine and catalase and 30% fetal bovine serum in low glucose Dulbecco’s modified eagle medium^45^. The cryopreservation protocol integrated with microchannel is shown in Fig. 10. For cryopreservation, the MSCs suspension was carefully injected into closed microchannel. The cell-seeded microfluidic device was then installed in 50 ml centrifuge tube (BD Biosciences) and stored at −80 °C. To evaluate recovery of cells and its metabolic activity, they were diluted gradually by adding pre-warmed media in each microfluidic chip. Each experiment was performed in triplicate.

#### Cell viability

The immediate post-thaw cell viability was measured by trypan blue dye exclusion assay. This process include addition of 10 μl (0.4% w/v) trypan blue solution into 10 μl cell suspension^46^. Cell viability was measured by counting cells in haemocytometer, where the stained cells were marked as dead cells. MTT assay of viable cells was used to measure the metabolic activity. The absorbance of the solution was recorded using UV-Visible spectrophotometer at 570 nm.

#### Cell adhesion number

We compared the number of adhered MSCs frozen on microchannel for 7 days with and without application of shear stress^47^. Microfludic experiment was carried out at very low shear stress (1 to 4.0 × 10^−3^ μbar). To bring uniformity in expression of focal point of adhesion in terms of expression of vinculin and actin filaments the experiment was carried out for 8 days. Furthermore, the experiment was followed according to the published literature^48^,^49^. The number of cells retained in the channel was evaluated. The number of cells was counted from four randomly selected microscopic cell images. Data were analyzed by means of standard deviations (more than three replicates were conducted in each experiment).

#### Cytoskeletal and focal point analysis

The cultured UCB-derived MSCs were fixed with 2% paraformaldehyde in PBS at 25 °C for 5 min. Cells were treated with 2% (w/v) bovine serum albumin and 1% (w/v) glycine followed by permeabilization with 0.5% Triton X-100 for 15 min. Samples were incubated with TRITC-Vinculin, FITC-Phalloidin and DAPI at 37 °C for 30 min in dark, and then observed under confocal laser scanning fluorescence microscope (Leica Microsystem, Germany).

To quantify focal point of adhesion, the acquired confocal images were further analyzed using threshold based image segmentation method. The acquired images consisted of two components namely the background and the foreground. Since the regions of interest were present in foreground, the backgrounds were removed for further processing and recognition of focal point adhesion. To accomplish this, a conventional frame difference method is used due to uniformly distributed intensity values of background in present case. Firstly, a reference mask similar to background of confocal images is generated. Then, the difference of background image and confocal image is determined^47^ which is shown in Fig. 3. After background removal, the red channel of the RBG color image was retained while green and blue channel were removed resulting in a gray scale image due to red color only. This gray scale image was converted in to binary image by using threshold method (Fig. 3). Pixel labeling was then carried out using 8-connectivity rule to determine various connected components in the gray scale image (Fig. 3).

#### Differentiation potential

Fresh and cryopreserved MSCs in microchannel were cultured in DMEM media supplemented with differentiation media. The osteogenic differentiation media comprised of dexamethasone (100 nM), β-glycerophosphate (10 mM), and L-ascorbate (0.2 mM). Adipogenic differentiation media comprised of indomethacin (50 μM), insulin (300 nM), dexamethasone (100 nM), and isobutylmethylxanthine (500 μM). The osteogenic and adipogenic potentials were measured by Alizarin red and Oil Red O staining.

#### Quantitative Real-Time polymerase chain reaction

RNA was isolated with Trizol reagent and quantified with nanodrop spectrophotometer. cDNA was synthesized using RNA-to-cDNA kit, and RT-PCR was performed in, 7500 Fast Real-Time PCR (Applied Biosystems, USA) using TaqMan Gene expression assay kit. Stemness marker genes such as NANOG (Hs01060663_m1), SOX-2 (Hs01053049_s1), OCT-4 (Hs04260367_g1), REX-1 (Hs01938187_s1) were used in the present study. Housekeeping gene used for normalization was GAPDH (Hs99999905_m1)^43^,^50^. The gene expression level of the control group (fresh MSCs or MSCs after induced shear stress) was normalized to 1. All the results were expressed as fold changes in gene expression relative to the control.

#### Statistical analysis

The experimental data were significantly analyzed statistically using Graphpad software (GraphPad Prism; Graphpad Software, Inc., San Diego, CA). The results are expressed as mean ± standard error. Comparative assessment of mean value among various factors was performed using ANOVA and unpaired t-test.

## Additional Information

**How to cite this article**: Bissoyi, A. *et al*. Enhanced cryopreservation of MSCs in microfluidic bioreactor by regulated shear flow. *Sci. Rep.* **6**, 35416; doi: 10.1038/srep35416 (2016).
